# Dataset on the use of 3D speckle tracking echocardiography in light-chain amyloidosis

**DOI:** 10.1016/j.dib.2018.04.013

**Published:** 2018-04-10

**Authors:** Antonio Vitarelli, Maria Teresa Petrucci, Silvia Lai, Carlo Gaudio, Lidia Capotosto, Enrico Mangieri, Serafino Ricci, Simone De Sio, Giovanni Truscelli, Federico Vozella, Mario Sergio Pergolini

**Affiliations:** Depts of Cardiology, Hematology and Medicine, Sapienza University, Rome, Italy

## Abstract

The dataset presented in this article is related to the research article entitled “Biventricular assessment of light-chain amyloidosis using 3D speckle tracking echocardiography: Differentiation from other forms of myocardial hypertrophy” (Vitarelli et al., 2018) [1], which examined the potential utility of left ventricular (LV) and right ventricular (RV) deformation and rotational parameters derived from three-dimensional speckle-tracking echocardiography (3DSTE) to diagnose cardiac amyloidosis(CA) and differentiate this disease from other forms of myocardial hypertrophy. The combined assessment of LV basal longitudinal strain, LV basal rotation and RV basal longitudinal strain had a high discriminative power for detecting CA. The data of this study provides more understanding on the value of LV 3DSTE deformation parameters as well as RV parameters in this particular cardiomyopathy.

**Specifications table**

**Table:** 

Subject area	*Medicine*
More specific subject area	*Cardiology (advanced echocardiography)*
Type of data	*Tables and figures*
How data was acquired	*The original raw data from three-dimensional data sets were analyzed on a separate software workstation (EchoPAC BT13, 4D Auto-LVQ, GE Vingmed-Ultrasound, Horten, Norway)*
Data format	*Analysed data presented*
Experimental factors	*Twenty-three patients with light chain amyloidosis were studied. Subjects with hypertrophic cardiomyopathy(HCM), systemic arterial hypertension(HTN), and athlete's heart(ATHL) were also studied (n=23 per group).*
Experimental features	*Patients were divided into five groups(AL-CA, HCM, HTN, ATHL, and controls). Echocardiographic parameters of LV-RV function (standard, 3DSTE) were compared between groups. The intraobserver and interobserver variabilities were determined as the absolute difference between each observer's value divided by the mean of both measurements and expressed as a percentage.*
Data source location	*Sapienza University, Rome, Italy*
Data accessibility	*Data is available with this article*

**Value of the data**

• The integration of the new 3DSTE echocardiographic techniques adds valuable information for the assessment of biventricular function in light-chain amyloidosis.
• LV basal longitudinal strain, LV peak basal rotation, and RV basal longitudinal strain appear independently associated with cardiac amyloidosis.
• A significant improvement in global chi-squared value is noted with RV 3D-strain parameters over only LV-3DSTE and conventional indices for detection of cardiac amyloidosis.
• These data help in analysing the use of LV and RV 3DSTE deformation parameters in amyloidosis cardiomyopathy.

## 1 Data

**Fig. 1:**
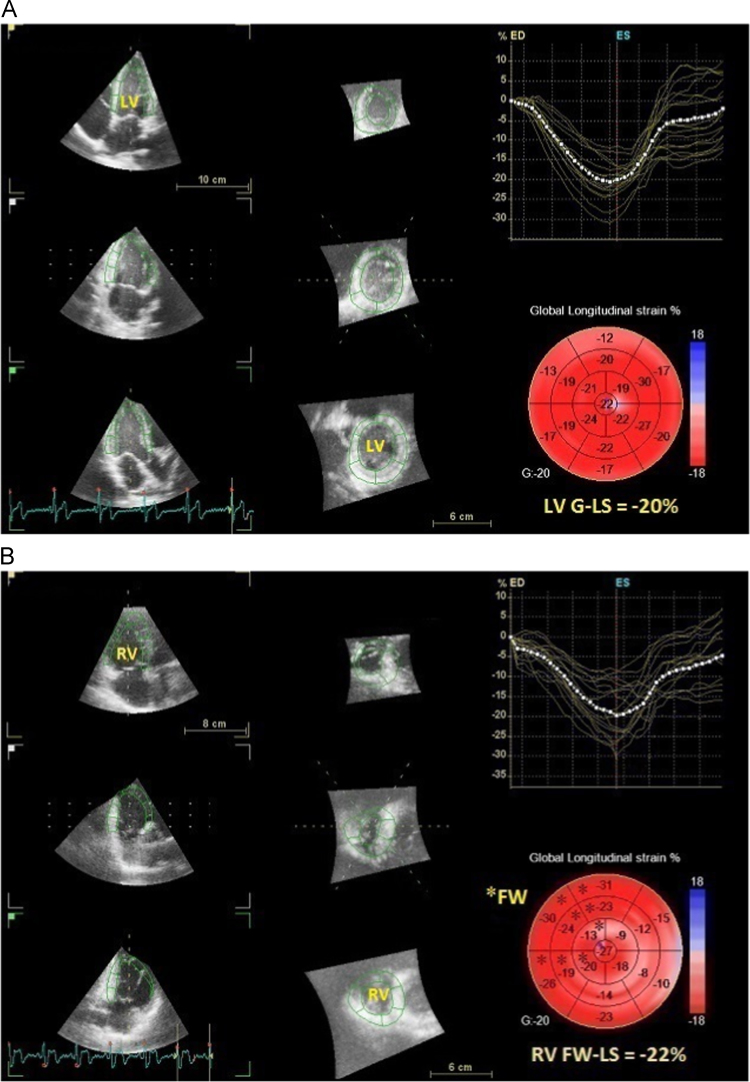
Representative LV and RV 3D strain images in normal controls. A. 3D LV speckle tracking multiplane view in a normal subject. 3D global longitudinal strain (LV G-LS) is −20%. B. 3D RV speckle tracking multiplane view in a normal subject. 3D global longitudinal strain (RV G-LS) is −20%. 3D global longitudinal strain of RV free-wall (RV FW-LS) was then calculated as −22% excluding septal segments.

**Fig. 2:**
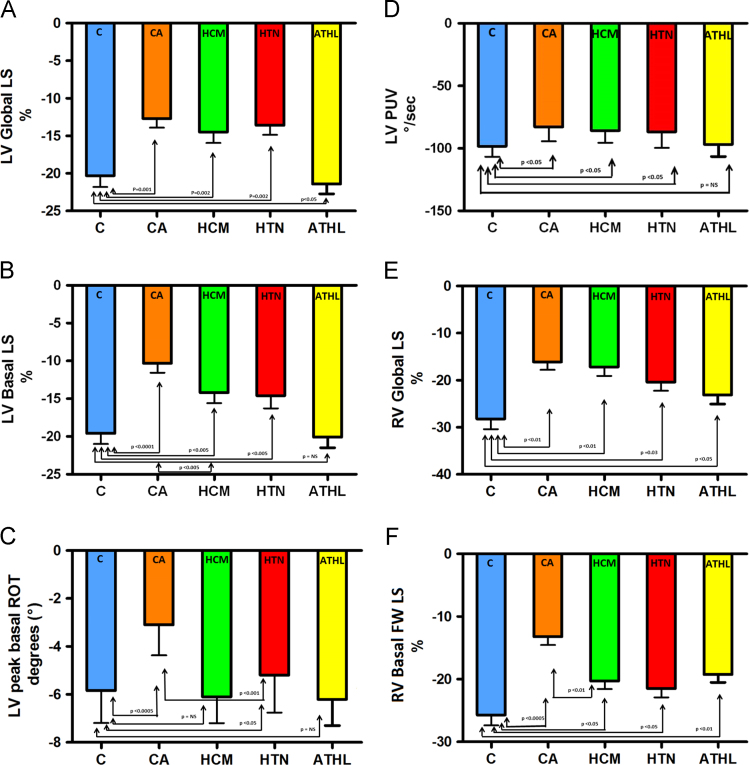
Bar graph depicting global and basal strain changes in left ventricular (LV) and right ventricular (RV) walls in cardiac amyloidosis(CA) patients compared to controls(C), patients with hypertrophic cardiomyopathy(HCM) and arterial hypertension(HTN), and athletes(ATHL). 1 A–C. CA patients show predominant decrease in LV basal longitudinal strain (LV basal LS) and LV peak basal rotation (ROT) compared to HCM and HTN patients. 1D**.** Peak untwisting velocity (PUV) is equally reduced in CA, HCM and HTN patients. 1E–F. CA patients show predominant decrease in RV basal free-wall longitudinal strain (RV basal FW LS) compared to HCM and HTN patients. Decrease in RV basal strain is also observed in athletes although less marked than CA patients.

**Fig. 3:**
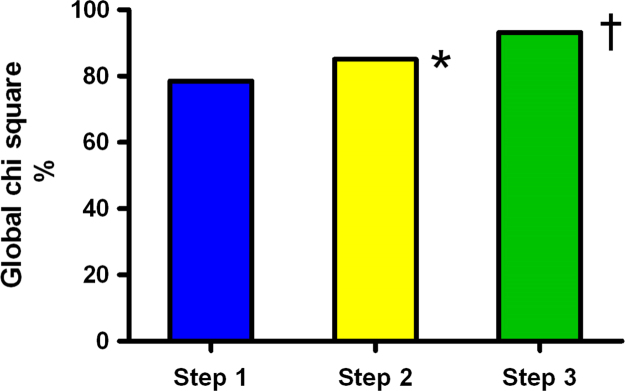
Incremental value of RV-3DSTE echocardiographic parameters in detecting CA over conventional and LV-3DSTE parameters. Step 1 included LV conventional parameters (DT and *E*/*E*_a_)+LV basal LS. Step 2 included DT+*E*/*E*_a_+LV basal LS+LV basal rotation. Step 3 included DT+*E*/*E*_a_+LV basal LS+LV basal rotation + RV FW basal LS. * Step1 vs step2: *χ*^2^ values 77.2 vs 84.6, *p*=0.003; † Step2 vs step3: *χ*^2^ values 84.6 vs 93.4, *p*<0.001.

The added material in this article extends and complements the data of the article entitled “Biventricular assessment of light-chain amyloidosis using 3D speckle tracking echocardiography: Differentiation from other forms of myocardial hypertrophy” (Vitarelli et al., 2018) [1]. Table 1 summarizes the conventional echocardiographic findings in patients, athletes and controls. Fig. 1 depicts representative LV and RV 3D strain images in normal controls. Fig. 2 includes bar graphs depicting global and basal strain changes in left ventricular and right ventricular walls in cardiac amyloidosis (CA) patients compared to controls, patients with hypertrophic cardiomyopathy and arterial hypertension, and athletes. Table 2 presents the univariate and multivariate analysis of parameters associated with CA. Fig. 3 shows bar graphs on the incremental value of RV-3DSTE echocardiographic parameters in detecting CA over conventional and LV-3DSTE parameters. Table 3 shows the results of receiver-operating characteristic curves in the overall population (92 participants) comparing several standard and 3DSTE echocardiographic parameters for their accuracy to predict CA.

**Table 1:** Conventional echocardiographic findings in patients, athletes and controls.

Parameters	Controls (*n*=23) Mean±SD	CA (*n*=23) Mean±SD	HCM (*n*=23) Mean±SD	HTN (*n*=23) Mean±SD	ATHL (*n*=23) Mean±SD	*P* value
**LV**						
LVED (mm)	48.7±4.2	49.2±4.8	50.1±4.3	53.4±3.9	51.3±4.5	NS
LVES (mm)	29.3±2.5	30.2±3.4	28.7±2.8	29.7±3.2	28.2±2.6	NS
IVST (mm)	9.5±1.7	14.8±1.7*, †	16.6±1.9	13.5±1.7	12.9±1.3	0.002
LVPWT (mm)	8.7±1.7	13.4±1.8	12.6±1.3	13.3±1.7	12.4±1.2	0.03
LVMI (g/m^2^)	79±19	145±29	141±36	139±27	129±26	0.02
LVEF (%)	65±5	62±7	67±6	63±4	65±7	NS
DT (ms)	224±49	151±43*, ‡	247±53	251±59	228±46	0.003
MV *E*_a_ (cm/s)	11.6±2.3	6.1±3.5	6.8±2.3	7.2±3.6	14.1±2.1	<0.05
MV *E*/*E*_a_	5.5±1.4	16.4±2.9*, d	13.5±2.6	12.4±1.7	2.8±0.9	0.004
**RV**						
RVED (mm)	22.6±2.3	24.7±2.6	23.3±2.4	25.3±2.1	24.3±2.2	NS
RVWT (mm)	4.1±0.7	5.9±0.9	5.7±0.6	5.1±0.8	4.5±0.6	0.01
RVSP (mmHg)	22±6	45±14	49±13	35±9	27±7	0.004
RVEDA (mm^2^)	16±5	21±4	17±5	22±5	20±5	NS
RVESA (mm^2^)	8±3	10±2	9±4	9±3	9±2	NS
RVFAC (%)	42±8	39±6	40±5	40±7	41±7	NS
TAPSE (mm)	23±5	14±5*, e	15±6	16±6	21±4	0.01
TV *S*_a_ (cm/s)	12.4±2.4	8.2±1.8	8.3±2.3	9.9±1.8	12.3±1.9	<0.05
TV *E*_a_ (cm/s)	11.2±2.4	9.6±2.7	8.5±2.2	8.9±2.1	12.9±2.1	0.05
TV *E*/*E*_a_	4.6±1.1	8.4±1.8	7.4±1.5	7.8±1.1	3.1±0.9	<0.01

DT=deceleration time of early filling; *E*=inflow early diastolic velocity; *E*_a_=annular early diastolic velocity; LVED=left ventricular end-diastolic diameter (Mmode); LVEDV=left ventricular end-diastolic volume (2D); LVEF=left ventricular ejection fraction (2D); LVES=left ventricular end-systolic diameter (Mmode); LVMI=left ventricular mass index (Mmode); LVPWT=left ventricular posterior wall thickness (Mmode); IVST=interventricular septal thickness (Mmode); MV=mitral valve; NS=not significant; RVED=right ventricular end-diastolic diameter (Mmode); RVEDA=right ventricular end-diastolic area (2D); RVESA=right ventricular end-systolic area (2D); RVFAC=right ventricular fractional area change (2D); RVSP=right ventricular systolic pressure; RVWT=right ventricular wall thickness (Mmode); *S*_a_=annular systolic velocity; TAPSE=tricuspid annular plane systolic excursion; TV=tricuspid valve.

* *p*<0.005 vs controls.

† *p*<0.005 vs HCM.

‡ *p*<0.01 vs HCM.

d *p*<0.05 vs HCM.

e *p*<0.001vs HTN.

**Table 2:** Univariate and multivariate analysis of parameters associated with CA.

	*Univariate analysis*		*Multivariate analysis*	
	*r*	*p*	*β*	*p*
Age	0.19	0.227		
Gender	0.17	0.346		
BMI (kg/m^2^)	0.12	0.513		
LVMI (g/m^2^)	0.31	0.038		
LV DT (ms)	0.35	0.026		
LV *E*/*E*_a_	0.29	0.032		
LV global LS (%)	0.54	0.013		
LV basal LS (%)	0.69	0.001	0.57	0.002
LV LS apical /basal ratio	0.62	0.003		
LV basal CS (%)	0.37	0.029		
LV peak twist (deg)	0.52	0.016		
LV peak basal ROT (deg)	0.61	0.002	0.42	0.003
LV PUV (°/s)	0.53	0.019		
RV global LS (%)	0.34	0.042		
RV FW LS (%)	0.41	0.013		
RV basal FW LS (%)	0.59	0.005	0.33	0.014

DT=deceleration time of early filling; BMI=body mass index; CS=circumferential strain; E=inflow early diastolic velocity; *E*_a_=annular early diastolic velocity; EF=ejection fraction; FW=free wall; LS=longitudinal strain; LV=left ventricle; LVEF=three-dimensional left ventricular ejection fraction (3DSTE); LVMI=left ventricular mass index (3DSTE); PUV=peak untwisting diastolic velocity; ROT=rotation; RV=right ventricle.

**Table 3:** Results of receiver-operating characteristic curves in the overall population (92 participants) comparing different standard and 3DSTE echocardiographic parameters for their accuracy to predict CA.

Variable	AUC	95% CI	*P* value	Cut-off	Sensitivity	Specificity
*LV*						
LV IVST	0.67	0.58–0.77	0.056	14.1 mm	68	59
LV DT	0.76	0.68–0.86	0.042	166 ms	69	78
LV *E*/*E*_a_	0.73	0.67–0.82	0.047	13.2	68	77
LV peak twist	0.81	0.74–0.93	0.043	9.8°	79	73
LV global CS	0.71	0.68–0.83	0.054	−23.1%	71	73
LV global LS	0.84	0.71–0.91	0.028	−13.7%	82	74
LV basal CS	0.80	0.70–0.94	0.033	−20.4%	81	79
LV LS apical/basal ratio	0.79	0.72–0.92	0.031	2.6	74	82
LV peak basal ROT	0.86	0.75–0.94	0.021	−3.9°	87	82
LV basal LS	0.89*	0.77–0.95	0.016	−11.7%	89	84
*RV*						
RVWT	0.61	0.58–0.86	0.055	6.4 mm	66	61
TAPSE	0.73	0.67–0.87	0.063	15 mm	77	63
RVFAC	0.61	0.59–0.86	0.076	39%	65	58
RVSP	0.54	0.52–0.81	0.079	46 mmHg	51	55
RV global LS	0.79	0.73–0.92	0.044	−16.6%	78	75
RV FW LS	0.83	0.77–0.97	0.022	−14.8%	85	76
RV basal FW LS	0.85	0.73–0.96	0.023	−13.3%	86	78
LV basal LS+peak basal ROT+RV basal FW LS†	0.93†	0.81–0.97	0.012	−11.7%, −3.9°, −13.3%	92	86

3D=three-dimensional; AUC=area under the curve; DT=deceleration time; CI=confidence interval; CS=circumferential strain; *E*=inflow early diastolic velocity; *E*_a_=annular early diastolic velocity; FW=free-wall; IVST=interventricular septal thickness; LS=longitudinal strain; LV=left ventricular; ROT=rotation; RVWT=RV wall thickness; RVFAC=RV fractional area change; RVSP=RV systolic pressure; TAPSE=tricuspid annular plane systolic excursion.

* *p*<0.001 compared to peak basal ROT.

† *p*<0.005 compared to LV basal LS, *p*<0.001 compared to RV basal FW LS.

## 2 Experimental design

Full experimental design has been described elsewhere [1], but in brief, twenty-three patients with light chain amyloidosis were studied. Subjects with hypertrophic cardiomyopathy(HCM), systemic arterial hypertension(HTN), and athlete's heart(ATHL) were also studied (*n*=23 per group). Twenty-three healthy subjects without cardiovascular disease and normal physical, electrocardiographic and echocardiographic findings were enrolled as controls.

## 3 Materials and methods

### 3.1 Echocardiography

Patients were examined in the left lateral decubitus position using a Vivid E9 commercial ultrasound scanner(GE Vingmed Ultrasound AS, Horten, Norway) with phased-array transducers. Grayscale recordings were optimized at a mean frame rate of ≥50 frames/s. Measurements of cardiac chambers were made by transthoracic echocardiography according to established criteria [2]. LV ejection fraction by modified biplane Simpson method and mass index were estimated [2]. LV deceleration time (DT) and right ventricular systolic pressure(RVSP) were determined [3], [4]. Mitral and tricuspid annular velocities(*S*_a_, *E*_a_, *A*_a_) were measured at the lateral corner of the mitral and tricuspid annulus on the transthoracic four-chamber views using spectral Doppler myocardial imaging. The mitral and tricuspid *E* to *E*_a_ ratio(*E*/*E*_a_) were used as indices for LA and RA pressure.

### 3.2 Three-dimensional speckle tracking echocardiography

Alignment was performed with presentation of four-chamber, two-chamber, and three-chamber apical views, as well as short axis views as previously described [5]. The original raw data from three-dimensional data sets were analyzed on a separate software workstation(EchoPAC BT13, 4D Auto-LVQ, GE Vingmed-Ultrasound, Horten, Norway). For the end-diastolic volumes, the operator placed one point in the middle of the mitral annulus plane and a second point at the LV apex generating an end-diastolic endocardial border tracing and including the papillary muscles within the LV cavity. For the end-systolic volumes, the same process was repeated in end-systole and acquisition of LV volumes and LVEF was obtained. The correct alignment of the endocardial contours during the cardiac cycle was checked to obtain the volume waveform. A second semiautomated epicardial tracking was made to delineate the region of interest for LV mass and strain analysis. The software provided segmental longitudinal, circumferential, radial, and 3D strain time curves, from which peak global strain and averaged peak strain at three LV levels (basal, midventricular, and apical) were obtained. Global longitudinal strain(LS), global circumferential strain(CS), global area strain(AS), global radial strain(RS), LS apical/basal ratio(average of apical segments divided by average of basal segments), and relative apical sparing(average of apical segments divided by average of basal and mid segments) were determined. Global area strain was calculated as the percentage decrease in the size of endocardial surface area defined by the vectors of longitudinal and circumferential deformations. LV twist was defined as the net difference(in degrees) of apical and basal rotation at isochronal time points [6], [7]. The 3D data sets were displayed as multi-planar reconstruction images in three short-axis slices from apex to base and apical four-chamber and two-chamber views. Adjustments were made in the multi-planar reconstruction images until the landmarks (mitral valve, LV apex, and aortic valve) were well positioned in each standard view. The software generated LV basal and apical rotation angles and LV twist time curves, from which peak basal rotation, peak apical rotation, and peak LV twist were obtained. LV torsion was normalized twist divided by LV ventricular diastolic longitudinal length between apex and mitral plane. Peak diastolic untwisting velocity and time to peak untwisting velocity were measured. Peak untwisting velocity(PUV) was determined as the greatest negative deflection following peak twisting velocity [8]. Time to peak untwisting velocity was the time to reach peak value measured from the QRS onset. Following a frame-by-frame analysis, a final 17-segment bull's-eye map of strain values was displayed (Fig. 1). Global strain values were automatically calculated by the software and were not determined in the presence of more than 3(of 17) uninterpretable segments.

Three-dimensional RV speckle tracking analysis was performed after imaging the RV chamber in apical views by using a 4 V phased array transducer with high volume rate(≥30 image frames/s) and six-beat acquisition with a methodology previously described [9], [10], [11], [12], [13]. The transducer was positioned at the modified apical position (more lateral than that in the standard apical view) for full RV coverage. RV analysis was similar to LV analysis, using placement of multiple reference points (instead of prefixed landmarks) all over the endocardial boundary to obtain 3D tracing of RV myocardium in long-axis and short-axis views. Three-dimensional global longitudinal strain of the whole RV was determined using the Echo PAC BT13 (Fig. 1). 3D global longitudinal strain of RV free-wall only (FW LS) was then calculated.

### 3.3 Statistics

Statistical analysis was performed using GraphPad Prism5 statistical software (San Diego, CA). Linear correlations, univariate and multivariate analysis were used for comparisons. Multiple comparisons of the data were analyzed through ANOVA assays with post hoc comparisons by Bonferroni test. Differences were considered statistically significant when the p value was <0.05. The incremental value of RV-3DSTE echocardiographic parameters in detecting CA over conventional echocardiographic variables and LV-3DSTE indices was assessed by calculating the *χ*^2^ increase of the multivariate model in logistic regression analysis.
