# Supplementary material for: Dataset on the use of 3D speckle tracking echocardiography in light-chain amyloidosis
Source: Data Brief. 2018 Apr 10;18:1239–46. doi: 10.1016/j.dib.2018.04.013 (PMC5996947; doi:10.1016/j.dib.2018.04.013)
Supplement: Supplementary file 1 — Supplementary material [file mmc1.docx]

Conflict of Interest

The authors report no relationships that could be construed as a conflict of interest.
